# Correlation between Optical Localization-State and Electrical Deep-Level State in In_0.52_Al_0.48_As/In_0.53_Ga_0.47_As Quantum Well Structure

**DOI:** 10.3390/nano11030585

**Published:** 2021-02-26

**Authors:** Il-Ho Ahn, Deuk Young Kim, Sejoon Lee

**Affiliations:** 1 Quantum-Functional Semiconductor Research Center, Dongguk University-Seoul, Seoul 04620, Korea; ihahn@dongguk.edu (I.-H.A.); dykim@dongguk.edu (D.Y.K.); 2 Division of Physics & Semiconductor Science, Dongguk University-Seoul, Seoul 04620, Korea

**Keywords:** InAlAs/InGaAs heterostructure, fermi-edge singularity, photoluminescence, deep level transient spectroscopy

## Abstract

The peculiar correlationship between the optical localization-state and the electrical deep-level defect-state was observed in the In_0.52_Al_0.48_As/In_0.53_Ga_0.47_As quantum well structure that comprises two quantum-confined electron-states and two hole-subbands. The sample clearly exhibited the Fermi edge singularity (FES) peak in its photoluminescence spectrum at 10–300 K; and the FES peak was analyzed in terms of the phenomenological line shape model with key physical parameters such as the Fermi energy, the hole localization energy, and the band-to-band transition amplitude. Through the comprehensive studies on both the theoretical calculation and the experimental evaluation of the energy band profile, we found out that the localized state, which is separated above by ~0.07 eV from the first excited hole-subband, corresponds to the deep-level state, residing at the position of ~0.75 eV far below the conduction band (i.e., near the valence band edge).

## 1. Introduction

InAlAs/InGaAs heterostructures have been widely studied in various aspects because of their vast potentials in both ultrahigh-speed electronic devices and highly-efficient optoelectronic devices that can operate in the infrared regimes. For instance, the high electron-mobility transistors [1,2,3,4] and the spin field-effect transistors [5] are feasible electronic devices that could be demonstrated on InAlAs/InGaAs heterostructures. In addition, the infrared photodetectors [6,7,8,9], X-ray detectors [10], terahertz (THz) quantum-cascade lasers [11,12], mid-infrared quantum-cascade lasers [13,14], and electro-optical modulators [15] are also typical examples that can open up a broad avenue toward the tangible optoelectronic applications of the InAlAs/InGaAs quantum well (QW) structures. When growing the epitaxial heterostructure, in general, both the alloy disorder and the layer-thickness fluctuation are inevitable because of the lattice mismatch in between the ultrathin heterojunction layers. This would eventually create interfacial defects, forming both the electrical deep-level states and the optical localization states inside the heterojunction. According to previous literature, those energy states may cause the Fermi-edge singularity (FES) phenomena [16,17,18,19,20,21], leading to the abnormal luminescence [21,22,23] and the anomalous carrier transport behaviors [24]. Furthermore, such an FES is known to significantly affect the device performances (e.g., decreased carrier mobility [25,26], increased kink-effect in resonant tunneling [27], cotunneling during the single-electron transport [28], increased electron-phonon coupling [29], increased electron-electron scattering in the photodiode [30], etc.). Based upon the above, one can conjecture that the FES-related states should be simultaneously represented with both the electronic band structures and the energy band diagram, respectively. To clarify this, thus, a comprehensive study on the relationship between the FES-related optical localization state and the electrical deep-level state is required to elucidate the essence of the correlation. However, the FES behaviors in the InAlAs/InGaAs QW systems still remain as a vivid debate.

Aiming at investigating how the FES behaviors correlate with the optical and the electronic energy band structures in the InGaAs/InAlAs QW, we thoroughly examined its FES-related localized-energy states by systematic analyses of temperature-dependent photoluminescence (PL) spectroscopy and Fourier-transform deep-level transient spectroscopy (FT-DLTS). Key procedures of this research can be described as follows: (1) calculation of the electronic band structure by using the self-consistent Schrödinger-Poisson equation [31,32], (2) observation of two abnormal PL features (i.e., appearance of the temperature-independent FES peak; and observation of all the red-shifted inter-subband transitions, comparing with the calculated band profile), (3) deconvolution of the low-temperature PL spectrum by using the phenomenological line-shape model [33], (4) extraction of the FES-related localization state; and verification of its corresponding red-shift in PL spectra, and (5) corroboration of the optical localization state by observing the electrical deep-level state through FT-DLTS. Herein, we describe and discuss the aforementioned theoretical and experimental results in detail.

## 2. Sample and Experimental Scheme

**Figure 1:**
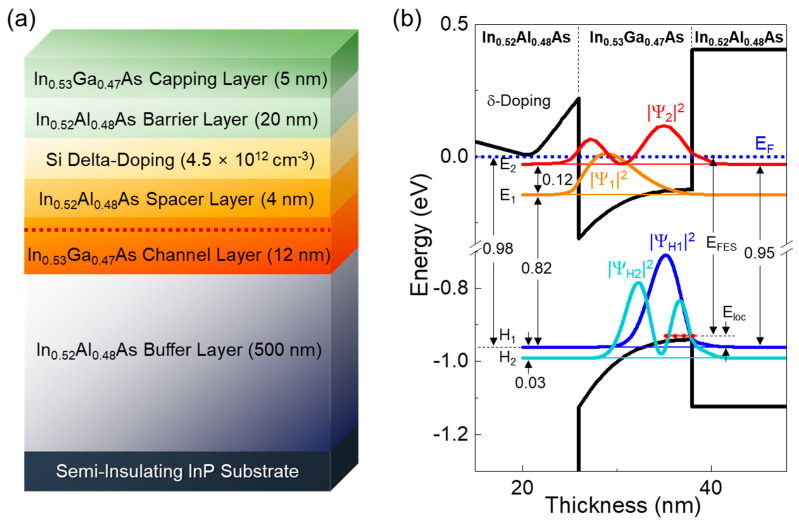
(**a**) Schematic of the In_0.53_Ga_0.47_As/In_0.52_Al_0.48_As quantum well (QW) structure. (**b**) Energy band profile of the In_0.53_Ga_0.47_As/In_0.52_Al_0.48_As QW structure calculated by Schrödinger-Poisson equations. The symbols of “|Ψ_1_|^2^ and |Ψ_2_|^2^” and “|Ψ_H1_|^2^ and |Ψ_H2_|^2^” in (**b**) denote the squared envelope functions for electrons and holes, respectively, at the quantum confined energy states.

Figure 1a schematically illustrates the In_0.53_Ga_0.47_As/In_0.52_Al_0.48_As QW structure that has been grown onto the (100) InP substrate by molecular beam epitaxy (MBE). Firstly, the InP surface was pre-cleaned by thermal heating at 520 °C in the MBE chamber. Then, the 500-nm-thick In_0.52_Al_0.48_As buffer layer was grown onto the (100) InP surface at 475 °C. Subsequently, the undoped In_0.53_Ga_0.47_As layer (12 nm) and the undoped In_0.52_Al_0.48_As spacer (4 nm) were grown onto the buffer layer at the same temperature. 

Next, the Si delta-doped sheet (4.5 × 10^12^ cm^−2^) and the undoped In_0.52_Al_0.48_As barrier layer (20 nm) were sequentially grown on the spacer layer. Finally, the 5-nm-thick cap layer of *n*-doped In_0.53_Ga_0.47_As was deposited on top of the sample in order to assist the Ohmic contact formation during the device fabrication steps [34].

To examine the optical properties of the sample, the temperature-dependent PL measurements were carried out at 10–300 K by using a home-built PL system [35,36], where the picosecond diode laser (λ_laser_ = 634 nm) and the time-correlated single-photon counter were equipped as an excitation source and a light emission detector, respectively. The excitation power density was fixed at 50 W/cm^2^ for all measurements. In order to extract the subband structure in the InGaAs/InAlAs QW, the PL spectra were analyzed by line-shape fitting in terms of the localization energy theorem [33]. In addition, the FT-DLTS measurements were also carried out to compare the energy values between the optical localization state with the electrical deep-level state. For the DLTS measurements, the Schottky contact was formed onto the top surface of the sample. Namely, after recess-etching of the *n*-In_0.53_Ga_0.47_As cap layer, the circular Ti/Au Schottky electrode (*ϕ*_dia_ ~ 300 μm) was formed onto the In_0.52_Al_0.48_As barrier layer. The Ohmic contact was formed onto the unrecessed *n*-InGaAs cap layer in the form of the ring electrode at the vicinity of the circular Schottky contact. After fabricating the Schottky diode structure, the DLTS measurements were performed with a 100 mV ac test signal at 1 MHz using a Bio-Rad DL8000 DLTS system (PhysTech, Moosburg, Germany).

## 3. Results and Discussion

### 3.1. Calculation of Energy Band Profile

Prior to the experimental characterization, we calculated the energy band diagram of the In_0.53_Ga_0.47_As/In_0.52_Al_0.48_As QW system to understand the quantum mechanical electric-energy structure of the fabricated sample. Figure 1b displays the energy band profile of the MBE-grown In_0.53_Ga_0.47_As/In_0.52_Al_0.48_As QW structure, which was calculated by using a Nextnano^3^ simulator (Nextnano GmbH, München, Germany) that had been programmed in terms of the transfer matrix function based on the self-consistent Schrödinger-Poisson equations [31,32]. From the calculated energy band profile, one may expect that several types of optical transitions are theoretically available because two excited electron-subbands (i.e., E_1_ and E_2_) and two excited hole-states (i.e., H_1_ and H_2_) coexist in the In_0.53_Ga_0.47_As/In_0.52_Al_0.48_As QW. In addition, the radiative optical transition between the Fermi level (E_F_) and H_1_ is also possible because of strong carrier population by δ-doping. The key transition parameters are listed in Table 1, where Δ*E_i_H_j_* (= *E_i_* − *H_j_*) denotes the energy gap between the quantum mechanical subbands.

### 3.2. Temperature Dependent PL Characteristics

**Figure 2:**
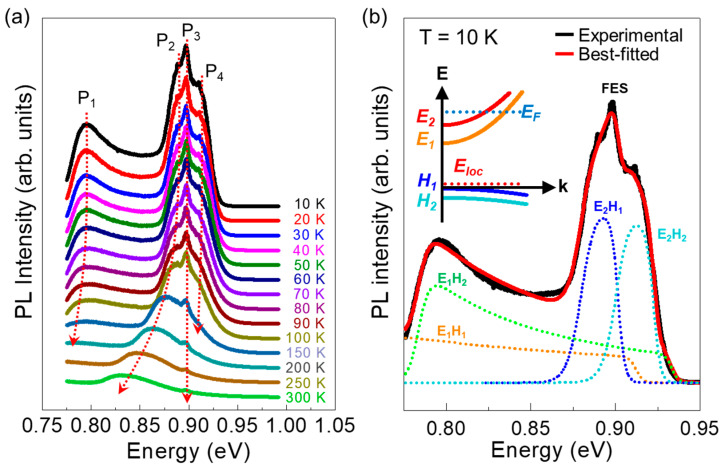
(**a**) Temperature-dependent PL spectra of the molecular beam epitaxy (MBE)-grown In_0.53_Ga_0.47_As/In_0.52_Al_0.48_As QW at 10–300 K. (**b**) Deconvoluted PL spectra at 10 K obtained by using the localization energy state-included line-shape fitting model. The inset illustrates the in-plane energy band scheme presented in the k-space.

After elucidating the energy band profile and the theoretically available optical transitions, we characterized the experimental emission properties of the MBE-grown InGaAs/InAlAs QW. Figure 2a shows the temperature-dependent PL spectra at 10–300 K of the sample. At 10 K, the InGaAs/InAlAs QW clearly exhibits four predominant PL peaks at P_1_, P_2_, P_3_, and P_4_, which correspond to E_1_H_2_, E_2_H_1_, FES, and E_2_H_1_ transitions, respectively, as discussed later. Here, it should be noticed that two abnormal PL features occur in the present InGaAs/InAlAs QW system. One is the temperature-independent behavior of the P_3_ peak; and the other is the approximately 70 meV red-shift of all the peaks, compared to the theoretically-available optical-transition energy values. We attribute the former to the FES phenomena because such an unusual temperature-independent PL feature could be explained by the many-body effect of the FES, arising from the Coulomb interaction between the photogenerated electron-hole pairs and the electrons in the Fermi sea [16,34]. The latter of the anomalously red-shifted inter-subband transition energies could also be ascribed to the FES nature because the FES causes a red-shift of the inter-subband emission due to the strongly-localized energy-state at the valence band edge of the InGaAs/InAlAs QW, as discussed below in Section 3.3.

### 3.3. Extraction of Inter-Subband Transition Energy Values via PL Line Shape Fitting

To clarify the above hypothesis, we carried out the line shape analysis of the low-temperature PL spectrum by using a localization energy state-included phenomenological fitting model [33]. According to this model, the total PL intensity can be represented as

(1) $$I\left(\hbar \omega \right)=\sum A_{ij}D\left(\hbar \omega \right)f_{ei}\left(\hbar \omega \right)f_{hj}\left(\hbar \omega \right)$$

where *A_ij_* is the transition coefficient including the inter-subband matrix elements; *D* is the broadened density-of-states step function; *f_ei_* and *f_hj_* are the Fermi distribution functions for electron and heavy-hole subbands, respectively. As shown in the deconvoluted PL spectra obtained from the above model (Figure 2b), the original PL spectrum could be well-resolved by four peaks. We could assign these four peaks as inter-subband transitions (*E_i_H_j_*); i.e., E_1_H_1_, E_1_H_2_ (= P_1_), E_2_H_1_ (= P_2_), and E_2_H_2_ (= P_4_), because of the following reasons. First of all, we here note that E_1_H_1_ was not labelled in Figure 2a because its exact peak had not emerged in the spectral range. Due to its explicit nature from the quantum mechanical calculation, however, the E_1_H_1_ peak should be included in the line shape fitting model. As summarized in the rightmost column in Table 1, consequently, the transition energies of E_1_H_1_, E_1_H_2_, E_2_H_1_, and E_2_H_2_ are red-shifted by ~70 meV from the theoretical values. Through integrating those four original *E_i_H_j_* transitions (i.e., dashed curves), additionally, the localization state-associated sharp FES (= P_3_) peak has clearly appeared in the best-fitted curve (i.e., red line). Furthermore, the three main peaks of E_2_H_1_, E_2_H_2_, and FES (i.e., stronger peaks) have an identical red-shift value of 70 meV. Accordingly, one can deduce that the inter-subband transition energies might be decreased parallelly by a single origin such as a localization energy state (*E_loc_*).

### 3.4. Extraction of Optical Localization State

Based upon the above concept, we can therefore draw the schematic band structure at the *k*-space (Figure 2b, inset). Considering the existence of *E_loc_*, we here assumed that the H_1_ state is the base level; and two heavy H_1_ and H_2_ bands contain the hole occupation probability. Then, the extracted parameters could be taken into account via the many-body effects, so-called FES phenomena [16,34]. The above assumption can be verified by three following results: (1) the magnitude of Δ*E_1_E_2_* (i.e., *E_1_* − *E_2_* = 120 meV) equals to each other in between theoretical and experimental cases; (2) the total carrier density (*n* ≈ 3.49 × 10^12^ cm^−2^) coincides with that obtained in our previous mobility spectrum analysis (*n* ≈ 3.43 × 10^12^ cm^−2^ [34]). Here, the total carrier density was estimated using the observed ”*E_F_* − *E_j_*” value by the following formula:

(2) $$n_{j}^{2D}=\frac{m_{j}^{*}}{\hbar^{2}\pi }k_{B}Tln\left[1+exp\left(\frac{E_{F}-E_{j}}{k_{B}T}\right)\right]$$

where $m_{j}^{*}$ is the effective mass; *k_B_* is the Boltzmann constant; *j* is 1 or 2 for the two subbands; *T* is the temperature; ℏ is the reduced Planck constant; *E_F_* is the Fermi energy, and *E_j_* is the *j*_th_ subband energy; (3) when comparing all the parameters, Δ*E_F_E_loc_* (= *E_F_* − *E_loc_* = *E_FES_* = 0.90 eV) is experimentally available, which signifies the localized state to locate at the valence band edge of the QW; then (4) Δ*E_loc_H_1_* (*= E_loc_* − *H_1_*) should be 70 meV, leading to the red-shift of the PL peaks by 70 meV because most of the excited carriers would transit into the localized state. As a result, the FES phenomena can take place between the localized state and the Fermi level.

### 3.5. Corroboration of Energetic Position for Localized State by DLTS

**Figure 3:**
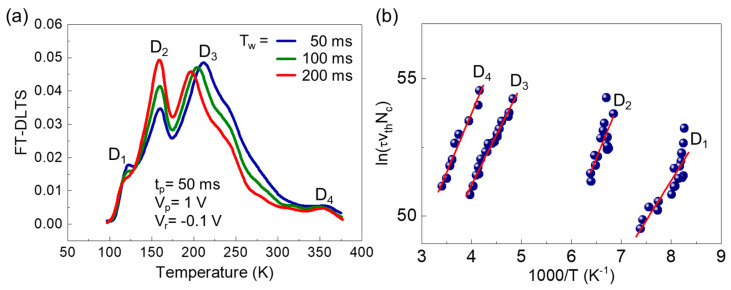
(**a**) Dependence of the Fourier-transform deep-level transient spectroscopy (FT-DLTS) signals on *T_W_* measured under *V*_r_ = −0.1 V, *V_P_* =1 V, and *t_P_* = 50 ms. (**b**) Arrhenius plots of FT-DLTS signals for D_1_, D_2_, D_3_, and D_4_.

The energetic position of the localized state can be further corroborated by observing its corresponding electrical deep-level state. For more clarity on the localized state position, we carried out FT-DLTS measurements [37]. Figure 3a shows the isothermal period (*T*_w_)-dependent FT-DLTS signals measured under the reverse bias voltage (*V*_r_ = −0.1 V) applied to the Schottky electrode, the filling pulse voltage (*V*_p_ = 1 V), the filling pulse time (*t*_p_ = 50 ms). The sample exhibits four DLTS signals of D_1_, D_2_, D_3_, and D_4_. Among them, one should focus on the peculiar phenomenon that, when varying *T*_w_ from 50 to 200 ms, the position of the D_2_ signal is fixed at ~150 K while its intensity becomes larger. Such an unusual *T*_w_-independent DLTS peak position is indicative of the localized state rather than band-like state [38]. In Figure 3b, the Arrhenius plots of the DLTS signals are shown. In the case of D_2_, the slope is steeper than the others. As summarized in Table 2, accordingly, the D_2_ possesses more substantial defect parameters. Particularly, in the present work, we focus on the energetic position of the D_2_ level (i.e., *E_C_* − *E_T_* ≈ 0.75 eV). Considering that In_0.53_Ga_0.47_As has a bandgap energy of ~0.75 eV [39,40,41,42], one can expect the localized D_2_ state to locate its position near the valence band edge. From all the above results, we can conclude that the energetic position of the located D_2_ state is consistent with that of the optical localization state.

## 4. Conclusions

We investigated the effects of the localized defect state on both the optical and the electrical properties of the In_0.53_Ga_0.47_As/In_0.52_Al_0.48_As QW structure. By using the phenomenological PL line shape fitting model, the optical localization energy, *E_loc_*, was determined to be 70 meV. Additionally, through both the optical and the electrical characterizations, the energetic position of the localized state was explicitly confirmed to exist at the valence band edge of the QW. Such a mutual correlation between optical and electrical localization states may lead to the strong confinement of photogenerated carriers. Because of the strong localization, the sample eventually exhibited two abnormal luminescence characteristics (i.e., an appearance of the strong FES persisting up to 300 K and a 70 meV red-shift of all the inter-subband transition energies in parallel).

## Figures and Tables

**Table 1:** Comparison of the physical parameters between the calculated (i.e., Schrödinger-Poisson equation) and the fitted values (i.e., localization energy-included line shape fitting).

Physical Parameters	Parameter Extraction Methods	
	Schrödinger-Poisson	PL Line-Shape Fitting
Δ*E_F_H_1_* (*= E_F_* − *H_1_*)	0.98 eV	-
Δ*E_F_H_2_* (*= E_F_* − *H_2_*)	0.95 eV	-
Δ*E_2_H_2_* (*= E_2_* − *H_2_*)	0.98 eV	~0.91 eV (Fitted)
Δ*E_2_H_1_* (*= E_2_* − *H_1_*)	0.95 eV	~0.88 eV (Fitted)
Δ*E_1_H_1_* (*= E_1_* − *H_1_*)	0.82 eV	~0.76 eV (Fitted)
Δ*E_1_H_2_* (*= E_1_* − *H_2_*)	0.85 eV	~0.79 eV (Fitted)
Δ*E_1_E_2_* (*= E_1_* − *E_2_*)	0.12 eV	0.12 eV (Estimated from Fitted Values)
Δ*H_1_H_2_* (*= H_1_* − *H_2_*)	0.03 eV	0.03 eV (Estimated from Fitted Values)
Δ*E_loc_H_1_* (*= E_loc_* − *H_1_*)	N/A	0.07 eV (Estimated from Fitted Values)
Δ*E_F_E_loc_* (*= E_F_* − *E_loc_ = E_FES_*)	N/A	0.90 eV (Best-Fitted)

**Table 2:** Defect parameters in the InGaAs channel determined from FT-DLTS measurements.

Defect Level	D_1_	D_2_	D_3_	D_4_
*E_C_* − *E_T_* (eV)	0.201	0.752	0.315	0.318
*σ* (cm^2^)	6.28 × 10^−15^	9.96 × 10^1^	1.36 × 10^−16^	1.10 × 10^−17^
*N_T_* (cm^−3^)	2.40 × 10^15^	7.88 × 10^15^	5.16 × 10^15^	3.44 × 10^15^
